# Micro(nano)plastics: an Emerging Burden for Human Health

**DOI:** 10.7150/ijbs.99556

**Published:** 2024-10-21

**Authors:** Isabella Donisi, Antonino Colloca, Camilla Anastasio, Maria Luisa Balestrieri, Nunzia D'Onofrio

**Affiliations:** Department of Precision Medicine, University of Campania Luigi Vanvitelli, Via L. De Crecchio 7, 80138 Naples, Italy.

**Keywords:** microplastics, nanoplastics, oxidative stress, toxic effects, human health threat

## Abstract

The escalation of plastic pollution represents a global environmental and health problem. Important toxic effects have been attributed to the increasing diffusion of microplastics (MPs) and nanoplastics (NPs) derived from the degradation of plastics. These particles have been ubiquitously observed in the environment, with humans being continuously exposed via ingestion, inhalation and skin contact. Nonetheless, the cellular homeostasis imbalance induced by micro- and nano- plastics (MNPs) in human health has been only recently shown, while most evidence and molecular mechanisms derived from studies *in vitro* and *in vivo* models. To date, the majority of available results testified the accumulation of MNPs in the cardiovascular, nervous, reproductive and digestive systems, and recently clear evidence about cardiovascular toxic effects of MNPs has been provided in humans. In this context, this review aims to provide a comprehensive update about the most recent studies reporting the effects of MNPs in different models, focusing on the available evidence in the main areas of study related to human health. Hopefully, this review will contribute to raise awareness about the toxicity and oxidative alteration exerted by MNPs, supporting the elaboration of new strategies to counteract plastic pandemics.

## Introduction

Plastics represent heterogeneous materials defined as “synthetic or heavily modified natural polymers” in a solid state and insoluble in water at 20°C 1. Their chemical composition is responsible of their environmental behavior, so as denser materials tend to deposit in the ocean bottom while lighter ones tend to float on the ocean surface 1. It is possible to distinguish three main phases in plastic life cycle: (i) production, resulting from energy-intensive and catalytic transformations, (ii) use, as single-use plastics, synthetic fibers and construction, and (iii) disposal. To date, plastic sustainable disposal, such as through recycling, is still below 10% globally, resulting in huge amounts of plastic wastes dispersed into the environment, accumulating in landfills, disrupting ecosystems and harming human's health 2.

Microplastics (MPs), fragments with a particle size of ≤ 5 mm, and nanoplastics (NPs), with a particle size of ≤ 1 μm, are hazardous forms of plastics ubiquitously found from the atmosphere to the hydrosphere 3. The global threat associated to the continuous release of MPs and NPs is promptly emerging, as of the accumulating evidence regarding the toxic effects on environmental and human health 4-6. Indeed, the occurrence of micro- and nanoplastics (MNPs) has been widely demonstrated in different ecosystems, representing an important source of harm due to their high biological incompatibility 7. However, given their ubiquitous presence, humans continuously come into contact with MNPs through inhalation, skin contact and ingestion 8, thus accumulating in blood, lungs, gastrointestinal and genitourinary tract, possibly causing multiple toxic effects 9. Moreover, also everyday kitchen activities, such as using plastic utensils and non-stick cookware, are a significant source of MPs ingestion, highlighting a pervasive daily source of exposure 10. Several studies assessed the toxic effects exerted by these small particles *in vitro* and *in vivo* models. MNPs exposure determined reactive oxygen species (ROS) formation as their main cellular effect, resulting in cell growth suppression, mitochondrial dysfunction and endoplasmic reticulum stress 11,12. The alterations of the redox state induced by MNPs has been attributed to extracellular and intracellular processes. Particularly, different factors, namely mechanical forces, temperature, light, chemicals and other variables can alter MPs surface molecule's structure leading to the formation of free radicals 13. Indeed, after MNPs are taken up by cells these particles are transported to lysosomes and then to mitochondria where they can alter the membrane potential, leading to increased ROS formation 13. Moreover, MNPs surface could adsorb harmful ligands as well as live microbial pathogens, functioning as a delivery system and amplifying their toxicity 14. Recent reports demonstrated a particle size-dependent absorption of endocrine disruptors pollutants, altering hormone receptors signaling and resulting in endocrine dysfunctions 6. Different studies are reporting toxic effects of MNPs at several levels, supporting their biological toxicity in different body districts. Interestingly, metagenomic analysis on gut microbiota evidenced that polystyrene MPs with a diameter ranging between 0.05-0.1 µm can affect fungal and bacterial composition and interaction, altering microbial metabolic pathways and inducing antibiotic resistance phenomena 15. *In vitro* human placental cells can internalize MNPs, possibly affecting pregnancy progression 16. MNPs exerted toxic effect on mice intestinal cells causing ROS-mediated apoptotic cell death and are responsible of cardiovascular performances impairment, hemolysis and increased thrombotic risk at macro- and micro- vascular level 17,18. NPs can be endocytated and induce autophagy activation and autophagosome formation, particularly in neurons, which are more susceptible to NPs exposure related stress and degeneration 19. Furthermore, human exposure to MNPs has been also identified as a cancer risk factors, exerting genotoxic and cytotoxic effects, according to the Agency for Toxic Substances and Disease Registry (ATSDR) reports 20. Even though more and more studies are evidencing toxic effects related to MNPs in various organs and tissues, most evidence available focuses on nervous, gastrointestinal, cardiovascular and reproductive system. Nonetheless, these systems have also been preliminarily investigated in human subjects, above all in the cardiovascular system. In this scenario, it is compelling to produce and spread evidence about the interplay between plastics consumption and health and environmental risk, as the base to restore the human right to live in a healthy environment, recognized by the United Nations 21. This review aims to provide the reader with the most recent and significant evidence about the effects of MPs and NPs in the nervous, reproductive, cardiovascular and digestive system, also highlighting the presence of studies in human models and to raise awareness about the emerging health and safety hazards to be faced by humanity.

## MNPs: what we know

Evidence indicate MNPs as widespread toxic pollutants, with environmental long-standing permanence. MNPs can present with different aspects, such as beads, fibers and films, and have different sizes and surfaces, determining their toxicity 22. Most of the available studies focused on the effects of MNPs on the nervous, reproductive, cardiovascular and digestive systems. As it follows, some of the most recent and relevant *in vitro* and *in vivo* evidence on the toxic effects of MNPs will be discussed.

### Effects of MNPs on the nervous system

**Figure 1:**
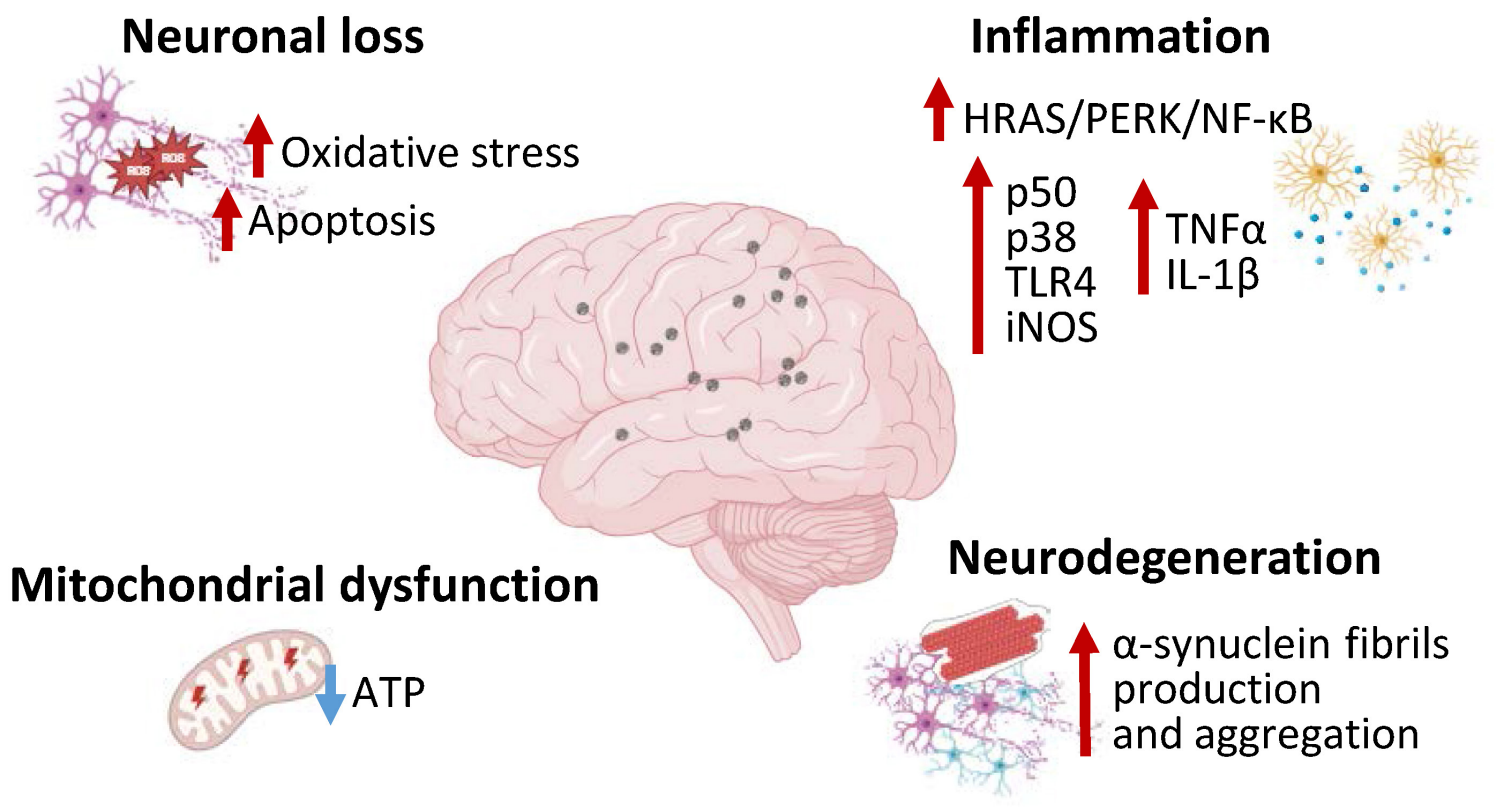
MNPs effects on the nervous system. Image representing the molecular pathways including inflammation, neurodegeneration, mitochondrial dysfunction, neuronal loss, activated in the nervous system during MNPs exposure. MNPs causes neural HRAS/PERK/NF-kB pathway activation and increased p50, p38, TLR4, iNOS, TNF-α, IL-1β expression. MNPs are also associated to neurodegeneration with increased α-synuclein production and aggregation along with mitochondrial dysfunction and neuronal loss. PERK—Protein kinase RNA-like endoplasmic reticulum kinase; NF-κB—Nuclear factor κB; TLR4—Toll-like receptor 4; iNOS—inducible nitric oxide synthase; TNF-α—tumor necrosis factor-α; IL-1 β—interleukin-1β.

Different studies investigated the effects of environmentally relevant MNPs on central nervous system, reporting their ability in causing excessive ROS and apoptosis in human nervous cell lines, with stronger effects detected in cortical neurons models 23,24 (**Figure 1**). Recently, evidence on the bioaccumulation of MNPs in the central nervous system has been provided via autoptic investigations on human brains and assessed via Pyrolysis Gas Chromatography-Mass Spectrometry 25. However, there is still no clear evidence reporting the consequences of MNPs accumulation on the human central nervous system. A pivotal role of MNPs-related neuronal cells degeneration has been attributed to oxidative stress. Specifically, MNPs exposure led to cortical neurons oxidative cell death and microbial pathogens can produce biofilms in MPs surface and exacerbate the neurotoxic effect 24.

Polystyrene MPs can accumulate in the brain and lead to anxiety-like behavior related to microglial activation and inflammatory response. In BV2 microglial cells polystyrene MPs treatment determined the activation of the HRAS/protein kinase R-like endoplasmic reticulum kinase (PERK)/nuclear factor (NF)-κB mediated inflammation and tumor necrosis factor-α (TNF-α) and interleukin (IL)-1β expression 26. Microglial N9 cells treated with NPs demonstrated p50, p38 and toll-like receptor (TLR)4 upregulation along with a notable increase in inducible nitric oxide synthase (iNOS) mRNA levels, evidencing the pro-inflammatory property of NPs 27 (**Figure 1**). Polystyrene NPs can induce ferroptosis and increased oxidative stress, related to altered glutathione metabolism, in brain microvascular cells, thus impairing blood brain barrier permeability and reducing zonula occludens 1 (ZO-1) protein expression 28. Moreover, polystyrene NPs determine downregulation of myelin basic protein (MBP) and myelin oligodendrocyte glycoprotein (MOG), thus damaging myelin structure, reducing oligodendrocytes number and leading to motor defects *in vivo* 29. Mice treated with polystyrene NPs exhibited impaired excitatory neurons energy metabolism, demonstrated by decreased adenosine triphosphate (ATP) cellular content and downregulated ATP-associated genes expression, as well as astrocytes and microglia inflammatory response, ultimately leading to a Parkinson's Disease-like phenotype 30. In this context, anionic NPs have been observed to promote α-synuclein protein fibrils formation and propagation via direct interaction with specific α-synuclein domains 31. NPs can be endocytated in a clathrine-dependent mode by neurons, causing lysosomal function impairment, reducing α-synuclein clearance 31. Furtherly, Jeong *et al.* described the capacity of NPs to exacerbate the extrapyramidal symptoms in C. Elegans and human cell models, testified by dopaminergic pauperization, locomotor dysfunction, and buildup of α-Synuclein aggregates 32 (**Figure 1**). In SH-SY5Y neuroblastoma cells NPs exposure resulted in cellular accumulation, with significant mitochondrial damages, altered morphology, reduced membrane potential and ATP production. Further investigations indicated that NPs mitochondrial disruption was associated to complex I interference and excessive mitophagy activation via AMPK/ULK1 pathway induction, thus leading to cell death 33. Overall, MNPs displayed multiple toxic effects on the nervous system, being correlated to impaired energy metabolism and neuronal dysfunction and degradation. The effects of MNPs on α-synuclein metabolism shed a new light on the correlation between the environment and neurodegenerative diseases, such as Parkinson's disease and other forms of dementia, suggesting their involvement in chronic degenerative diseases. Most studies concerning the effects of plastics on the nervous system have focused on the role of NPs more than MPs, which would suggest a major susceptibility of neurons and other neural cells to NPs. It could be hypothesized that *in vivo* systems NPs can penetrate brain barrier more efficiently than MPs, in virtue of their smaller size, and exert toxic effects. In addition, NPs, compared to MPs, could enter neuronal cells with peculiar, efficient but still unknown mechanisms and alter cellular specific signaling pathways 19. The plausible mechanism of action in the nervous system could be related to excessive ROS production induced by MNPs in neurons and other neural cells determining endoplasmic reticulum stress, mitochondrial damages, activation of NF-κB inflammatory pathway, damage in myelin structure, leading to ferroptosis or mitophagy cell death process. Nonetheless, further studies are required to clarify the mechanisms of MNPs toxicity on the nervous system.

### MNPs on the reproductive system

Among the toxic effects exerted by MNPs, the impact on reproduction, including male and female reproductive system as well as embryonal development, is alarming 34,35. Montano *et al.* detected MPs in the ovarian follicular fluid of women undergoing assisted reproductive treatment. Their study revealed a significant correlation between MPs concentration and levels of follicle-stimulating hormone (FSH), as well as weaker associations with body mass index (BMI), age, and estradiol 36. Moreover, a recent study conducted on 22 female patients evidenced the accumulation of MPs, including polyurethane, polypropylene, polystyrene and polyethylene, in human endometrium. Even though this result suggests the existence of toxicity on female's reproductive system, the effects are still to be assessed 37. In addition, MNPs have also been identified in the vagina, which has been characterized with a thinner and more permeable stratum corneum, making it more susceptible to their effects. For instance, Pontecorvi *et al.* utilized a human vaginal keratinocyte cell model to reveal that internalized MNPs impaired the expression of junctional and adherence proteins, disturbed the organization of the actin cortex and influenced genes involved in oxidative stress signaling pathways and miRNAs crucial for vaginal epithelial barrier function 38. In males, the investigation of 6 testis and 30 semen human specimens assessed the accumulation of plastic particles in the reproductive system 39. A similar study evaluated the concentration of MPs in human testis, with polyethylene being the most abundant, and the presence of an inverse correlation between testis weight and MPs concentration 40. The accumulation of MPs has also been reported in prostate samples of 6 out of 12 patients examined 41. Additionally, the study carried out by Chen *et al.* revealed high MNPs concentration and significant impairment of human semen function. Indeed, polystyrene NPs were capable of penetrating and damaging sperm cells 42. Finally, a recent study assessed the accumulation of MPs in human placental samples, with polyethylene being the most prevalent 43. Indeed, in the study carried out by Amereh *et al.*, increased exposure to MPs was associated to lower birth weight, reduced length, smaller head circumference, in intrauterine growth restriction (IUGR) pregnancies compared to those classified normal pregnancies 44. Current evidence demonstrated MNPs accumulation via the blood-placental barrier during pregnancy and trough the breast milk during nursing in different organs, leading to a dysregulated metabolism and impaired immune and reproductive function 45,46. Given the bioaccumulation of MNPs in human reproductive system, several studies are being conducted to evaluate their toxicity *in vitro* and *in vivo* models.

**Figure 2:**
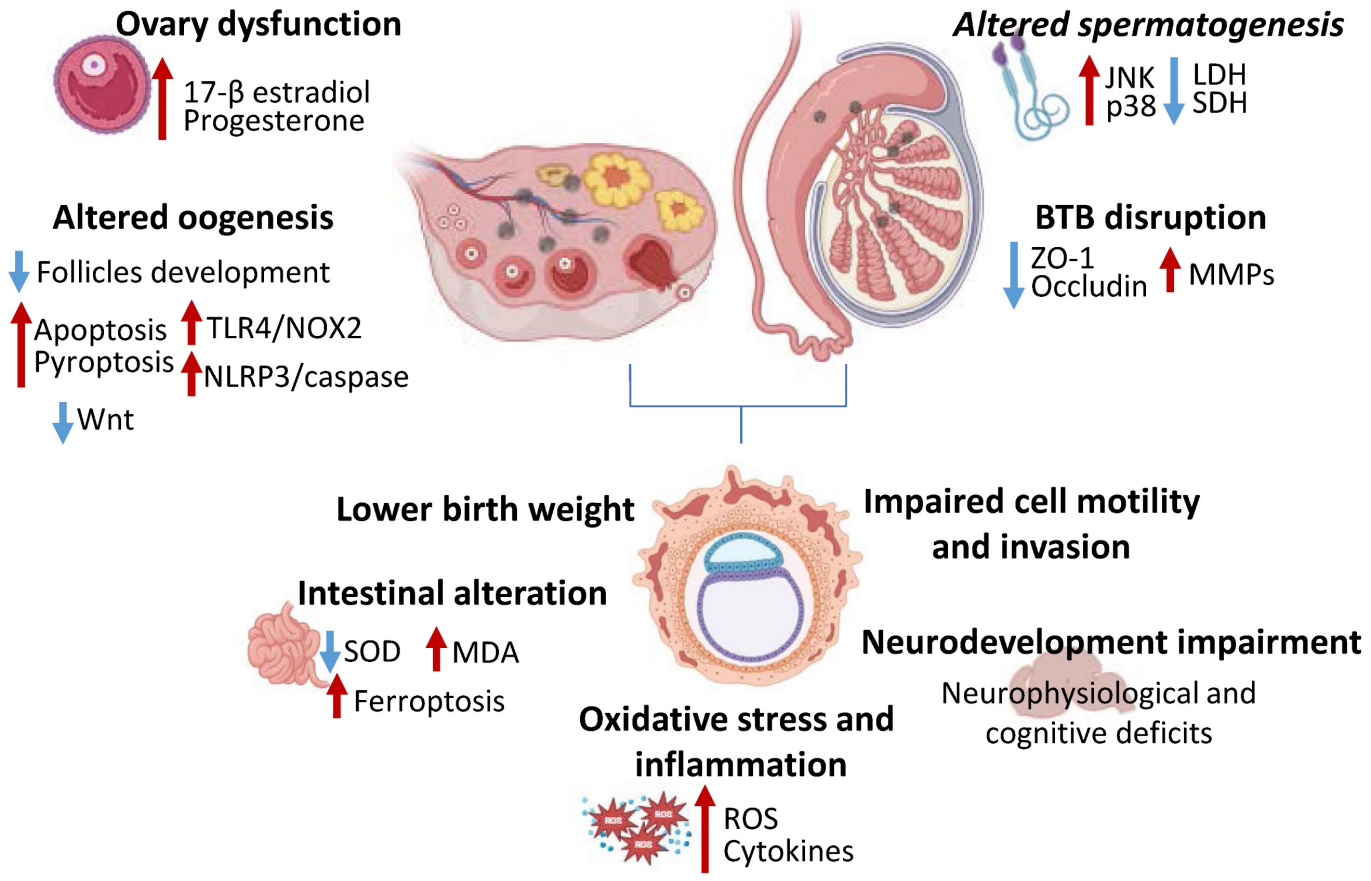
Effects of MNPs on reproduction. Image showing how MNPs impair ovary-related sexual hormones secretion, reduce follicles development, increase TLR4/NOX2 and NLRP3/caspase pathways, thus triggering apoptosis and pyroptosis and reduce Wnt signaling. MNPs alter spermatogenesis via JNK and p38, and decreasing LDH and SDH activity, moreover, disrupt blood-testis barrier, reducing ZO-1 and occluding expression and increasing MMPs. MNPs affect embryonal development being associated to lower birth weight, impaired cell motility and invasion, altering neural and intestinal development and increasing oxidative stress and inflammation. TLR4—Toll-like receptor 4; NOX2—NADPH Oxidase 2; NLRP3— NOD-like receptor protein 3; JNK—c-Jun NH2-terminal kinase; LDH—Lactate dehydrogenase; SDH—Succinate dehydrogenase; ZO-1—zonula occludens 1; MMPs—matrix metalloproteinases; SOD—Superoxide dismutase; MDA—Malondialdehyde; ROS—reactive oxygen species.

Treatment with polystyrene MPs in HTR-8/sVneo human placenta cell lines led to increased ROS and inflammatory cytokines production and reduced cell survival 47 (**Figure 2**). Moreover, MPs treatment impaired cell motility and invasion ability, negatively affecting the placentation process 47. Polystyrene NPs exposure altered cell membrane integrity and induced autophagy initiation and autophagosome production, along with mitochondrial damage, in human umbilical vein endothelial cells (HUVECs) 48,49. It has been observed that maternal administration of polystyrene NPs in mice impaired progeny nervous system development and led to neurophysiological and cognitive deficits 50. Tang *et al.* demonstrated that mice exposed to polystyrene NPs during gestation displayed lower birth weight and higher adult weight compared to non-NPs exposed mice 51. Moreover, it has been shown that the intake of MPs during the prenatal and early post-natal period can provoke neurodevelopmental disorders in mice offspring caused by a diminished dopamine transporter protein in the brain, or dysregulated gene expression 52. Relevant findings indicate that NPs accumulation in the blood-brain barrier is significantly higher during the early embryonic period due to the underdeveloped fetal blood-brain barrier in the early stages of life, which permits NPs to penetrate and potentially induce neurological dysfunction 45,46. An* in vivo* study on maternal rats revealed neurological developmental disorders resulting from the exposure of fetal brain tissue to NPs during early developmental stages 45. Another study discovered that NPs transferred to fetuses during gestation affect the function and composition of neural cells and alter the brain histology of neural stem cells cultured from offspring 53. Moreover, NPs exposure was associated to small intestine histological alterations, which were related to impairment of superoxide dismutase (SOD) and malondialdehyde (MDA) levels and activation of ferroptotic pathway 47 **(Figure 2).** Also, recent research indicated that offspring's hearts are more susceptible to damage from MNPs compared to the fully developed adult hearts 54. Indeed, a significant reduction in mitochondrial membrane potential and an altered cellular metabolism, which coincided with a marked decrease in electrophysiological activity during the early stages of contractility has been observed in *in vitro* experiments conducted on neonatal rat ventricular cardiomyocytes 46,55.

Polystyrene MPs exposure in male mice increased testicular oxidative stress and induced the activation of c-Jun NH2-terminal kinase (JNK) and p38 mitogen-activated protein kinase (MAPK) pathways, which resulted in decreased sperm number and motility, as well as suppressed metabolism-related enzymes lactate dehydrogenase (LDH) and succinate dehydrogenase (SDH) activity 56. Moreover, a synergic effect of polystyrene NPs and lipopolysaccharide (LPS) has been detected, demonstrated by reduced sperm count, lower testosterone levels and steroidogenic acute regulatory (StAR) expression levels, along with severe inflammatory response and oxidative stress 57. Ma *et al.* assessed the toxic effect of dibutyl phthalate-charged NPs on the blood-testis barrier in mice and *in vitro*. In this study, NPs induced spermatogenesis impairment and disrupted blood-testis barrier, testified by decreased sperm quality, reduced ZO-1 and occludin and increased matrix metalloproteinases (MMPs) expression 58 (**Figure 2**). Furthermore, in toxicological studies on rodents and cell cultures MNPs have been shown to produce considerable damages, including abnormal sperm structure and reduced quality 59. In addition, mice exposed to MPs and NPs demonstrated an impairment of sperm motility, linearity and velocity, especially when exposed to 25-30 nm and 1-5 µm particles 60.

On the other hand, studies demonstrated that exposure to polystyrene MPs can lead to ovary dysfunction with reduced follicles development and embryos number and deleterious impact on female fertility and reproduction 61,62. For instance, recent studies elucidated the harmful effect of MPs and NPs exposure on the ovarian tissue which can lead to a lower number of developing follicles, to a reduced number and diameter of small uterine arteries provoking a deleterious effect on female fertility and ovarian reserve 63,64,65. MPs induced granulosa cells apoptotic and pyroptotic cell death, activating NOD-like receptor protein 3 (NLRP3)/caspase signaling and impairing Wnt pathway, as well as inducing TLR4/ NADPH Oxidase (NOX)2 pathway activation and uterine fibrosis 65 (**Figure 2**). Indeed, this aspect has also been confirmed in different works where the increased ROS levels is related to the reduction of antioxidant enzymes following MNPs exposure 63,66. The effects on NPs on the reproductive system have also been investigated on swine granulosa cells, reporting increased cell proliferation with disrupted 17-β estradiol and progesterone secretion and increased oxidative stress, deeply altering ovary follicles development and function 62 (**Figure 2**).

To this end, evidence on the effects of MNPs on the reproductive system appear to be extremely polymorphic, considering the possible effects on future generations occurring since the moment of fecundation. Different studies already indicate the accumulation of MPs and NPs in both human male and human female genital tracts, as well as in fetal tissues, and several studies in mice also evidenced MNPs involvement in developmental alterations, including central nervous system development.

Undoubtedly, the ability of MNPs to induce oxidative stress in the reproductive system correlates at molecular level with apoptotic and pyroptotic cell death, via NLRP3/caspase signaling, with the activation of JNK/MAPK pathways, along with mitochondrial damage, altered cell membrane integrity and autophagy initiation and autophagosome production. These cellular events alter signaling pathways pivotal to an adequate function of these tissues could be correlated to hormonal dysfunction and increased germinal tumor development. Possibly, further studies and trials will allow to assess these effects in human models and possibly find countermeasures.

### Effects of MNPs on cardiovascular system

**Figure 3:**
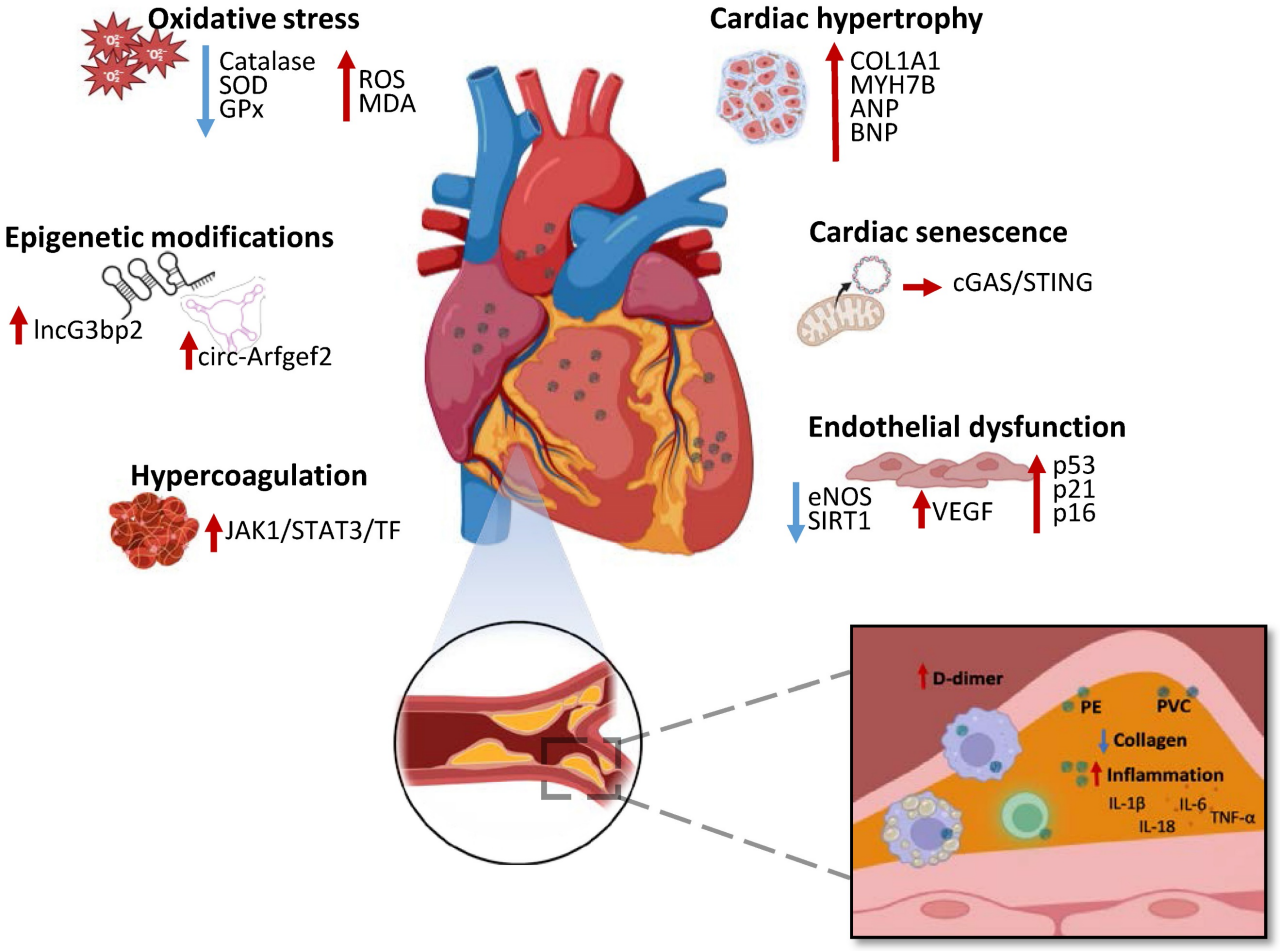
Effects of MNPs on the cardiovascular system and representation of human atherosclerotic plaque with MNPs. MNPs determine oxidative stress with increase of ROS and MDA levels along with decrease of catalase, SOD and GPx activity, cardiac hypertrophy with COL1A1, MYH7B, ANP and BNP upregulation, cardiac senescence with mitochondrial DNA leakage and activation of cGAS/STING pathway, alteration of cardiac epigenetics, endothelial dysfunction associated to eNOS and SIRT1 downregulation and VEGF, p53, p21 and p16 upregulation and an hypercoagulative state with JAK1/STAT3/TF pathway activation. Atheromas from patients with high levels of PE and PVC show decreased collagen, increased proinflammatory cytokines IL-6, IL-1β, IL-18, TNF-α, along with increased inflammatory cells and increased D-dimer plasmatic levels. ROS—Reactive oxygen species; MDA—Malondialdehyde; SOD—Superoxide dismutase; GPx—Glutathione peroxidase; COL1A1—Collagen type I alpha 1; MYH7B—Myosin heavy chain 7B; ANP—atrial natriuretic peptide; BNP—Brain natriuretic peptide; cGAS—Cyclic GMP-AMP synthase; STING—Stimulator of interferon genes; eNOS—Endothelial nitric oxide synthase; SIRT1—Sirtuin 1; VEGF—Vascular Endothelial Growth Factor; JAK1—Janus kinase 1; STAT3—Signal transducer and activator of transcription 3; TF—Tissue factor; PE—Polyethylene; PVC—Polyvinyl chloride; IL-1 β—interleukin-1β; IL-6— interleukin-6; IL-18— interleukin-18; TNF-α—tumor necrosis factor-α.

The effects of MNPs have been recently investigated and demonstrated in the cardiovascular system, being associated to different alterations widening from cardiac dysfunction to atherosclerosis 22. In this scenario, Marfella *et al.* is a landmark study linking MNPs to serious human health problems (NCT05900947) 67. In this observational study, 304 patients undergoing carotid endarterectomy were enrolled, with 257 patients who completed the study follow-up. The excised atheromas were examined and the level of polyethylene and polyvinyl chloride MNPs was quantified. The results of enzyme-linked immunosorbent assays and immunohistochemistry evidenced increased IL-18, IL-1β, IL-6 and TNF-α, decreased collagen and increased cluster of differentiation (CD)3 and CD68 plaque content (**Figure 3**). During the follow-up, the number of myocardial infarctions, stroke and any-cause death was compared in patients who displayed MNPs-associated plaques versus MNPs clean plaques patients. Notably, the results of the study evidenced higher mortality and morbidity rates in patients whose atheromas were infiltrated with MNPs (NCT05900947) 67 (**Figure 3**). Significantly, a successive human-based study, analyzing thrombi collected by 30 patients undergoing stroke, myocardial infarction and deep vein thrombosis related surgery, provided sustaining evidence detecting MNPs, above all polyethylene, in 80% of samples, which were associated to higher patients D-dimer levels and greater disease severity 68. Moreover, Liu *et al.* evaluated the concentration of MPs in coronary and carotid arteries plaques and in the non-atherosclerotic aorta. The concentration of MPs was reported to be significantly higher in the atheroma associated arteries specimen compared to non-pathologic aorta, supporting the role of plastic particles in cardiovascular atherosclerotic 69.

Nonetheless, the effects of MNPs on human cardiovascular system still need to be comprehensively explained, with numerous studies *in vitro* and *in vivo* suggesting an even vaster toxicity. Mice- and human-derived cardiac organoids exposed to different concentration of MPs demonstrated increased oxidative stress, inflammation, apoptotic cell death and collagen accumulation, which resulted associated to hypertrophic-related genes expression, such as myosin, heavy chain 7B (MYH7B), atrial natriuretic peptide (ANP), brain natriuretic peptide (BNP), Collagen, type I, alpha 1 (COL1A1) 70 (**Figure 3**). In mice models, administration of MPs determined upregulation of circular RNA circ-Arfgef2 and long non-coding RNA lncG3bp2 N6-methyladenosine (m6A) methylation modifications and expression, which has been suggested as a potential mechanism of MPs cardiotoxicity 71 (**Figure 3**). Polyethylene terephthalate MPs administration to mice led to capillary congestion, myocardial fiber breakage and fibrosis, associated to cardiomyocytes apoptotic cell death 72. Contextually, *in vitro* myocardial cells treated with MPs demonstrated ROS accumulation, elevated MDA level with decreased catalase, SOD and glutathione peroxidase (GPx) decreased activity 72 (**Figure 3**).

NPs long term exposure on cardiac cells *in vitro* and *in vivo* models determined cell senescence and dysfunction via mitochondrial destabilization with leakage of mitochondrial DNA (mtDNA) in the cytoplasm and activation of cyclic GMP-AMP synthase (cGAS)/Stimulator of interferon genes (STING) pathway 73 (**Figure 3**). Endothelial cells treated with NPs underwent premature senescence, demonstrated by the observation of senescence markers expression, such as p53, p21 and p16. Particularly, NPs related senescence has been associated to downregulated endothelial nitric oxide synthase (eNOS), impairing normal vasal motility, as well as increased NOXs and suppressed sirtuin (SIRT)1 expression 74 (**Figure 3**). Aortic endothelial cells treated with NPs displayed disrupted redox state associated to increased metabolic activity and Vascular Endothelial Growth Factor (VEGF) expression, negatively affecting vasal homeostasis 75 (**Figure 3**). Wang *et al.* demonstrated that polystyrene NPs mice exposure results in vascular endothelial damage with the activation of janus kinase 1 (JAK1)/Signal transducer and activator of transcription (STAT)3/tissue factor (TF) signaling and dysfunctional prothrombotic state 76 (**Figure 3**). Mice and RAW264.7 macrophages exposed to polystyrene NPs exhibited lipid accumulation, altered redox status and inflammation, associated to atherosclerotic plaques formation 77. In conclusion, MNPs can affect multiple pathways related to cardiac fibrosis and dysfunction, endothelial and coagulative impairment, along with increased atheroma formation, suggesting their molecular role in determining negative cardiovascular events. The recent landmark study published by Marfella *et al.* contributed to confirm the existence of toxic effects exerted by MNPs in humans, raising awareness all over the world. In addition, MNPs entering the cardiovascular system, could be accumulating also in cardiomyocytes and platelets, implying the presence of other pathways determining negative cardiovascular outcomes related to MNPs in humans. Nonetheless, the molecular mechanisms responsible of these effects are still to be defined with novel studies *in vivo* and *in vitro*. Possibly, upon accumulation in cardiac tissue, MNPs trigger oxidative stress trough ROS production, activating the NLRP3 inflammasome and JAK1/STAT3 pathways, which promote inflammation, endothelial dysfunction, thrombosis and atherogenesis, ultimately leading to increased risk of myocardial infarction and stroke.

### Effects of MNPs on the digestive system and gut microbiota

The effects of MNPs exposure on human digestive system have not been clarified yet, with no trials or clear evidence indicating specific toxic mechanisms in human models. Nonetheless, accumulating studies have clearly reported the ability of MNPs to influence and modify gut microbiota composition with effects at a local, as well as systemic, level 19. For instance, a recent study indicated that humans highly exposed to MPs exhibited abundance of pathogenic Klebsiella and Helicobacter and reduced beneficial Bacteroides in the nose compared to lowly exposed subjects. Furtherly, humans overexposed to MPs displayed disease associated Bifidobacterium, Streptococcus, and Sphingomonas abundance along with reduced healthy Ruminococcus Torques group, Dorea, Fusobacterium, and Coprococcus 78. The effects of MNPs on the gut microbiota and digestive system health have been investigated in mice models. Mice exposed to polyethylene MPs displayed alterations in gut microbial number, species and diversity. Particularly, MPs exposure determined an increase in Staphylococcus and reduction of Parabacteroides abundance and was correlated to increased IL-1α serum levels, with intestinal inflammatory aspects and higher TLR3, activator protein (AP)-1 and interferon regulatory factor (IRF)5 expression 79. Furtherly, Jing *et al.* observed an association between abnormal gut microbiota and bone marrow alterations, with decreased colony-formation, self-renewal and differentiation abilities and increased lymphocyte abundance in mice treated with MPs and NPs 80. Moreover, long-term MPs administration or fecal transplantation of microbiota from MPs-treated mice negatively affected bone marrow health and was associated to reduced Rikenellaceae and hypoxanthine intestinal content and subsequent decreased survival and proliferation of hematopoietic stem cells via inhibition of hypoxanthine-guanine phosphoribosyltransferase (HGPRT)/Wnt signaling 81. Interestingly, human patient who underwent hematopoietic stem cells transplantation exhibited a negative correlation between MPs levels and transplanted patients' survival time, as well as a positive correlation between patient survival and Rikenellaceae and hypoxanthine intestinal content 81. Zhang *et al.* indicated that the hepatic damage correlated to MPs could be attributed to microbial alterations. Mice treated with MPs exhibited systemic and hepatic inflammation and fibrosis along with intestinal barrier disruption, colonic inflammation accompanied by decrease in probiotics Akkermansia, Mucispirillum, and Faecalibaculum and increased pathogenic Tuzzerella. Particularly, the elimination of gut microbiota or the administration of an antibiotic cocktail reduced MPs-associated systemic inflammation as well as hepatic inflammation 82. Other studies have also evidenced direct toxic effects of MNPs on the gastrointestinal system independently from gut microbiota. Mice models exposed to polypropylene MPs for 4 weeks demonstrated damaged and dysfunctional hepatic structure 83. Furtherly, the hepatotoxic effect of MPs has been related to activation of pyroptotic pathway, activation of NLRP3 inflammasomes and apoptosis associated speck-like protein (ASC) and increased lipid peroxidation, accompanied by ferroptosis trigger 83. MPs could determine hepatocytes nuclear and mitochondrial DNA damage with subsequent activation of cyclic cGAS/STING/NF-κB pathway and production of pro-inflammatory cytokines, finally leading to liver dysfunction and fibrosis 84. Mice and intestinal organoids exposed to benzo [a] pyrene-loaded polystyrene MPs displayed increased Notch signaling activation, related to the increased ROS production. The overactivation of Notch in these models resulted in disrupted colonic mucosal barrier and tight junctions' injury, with increased inflammation, autophagy and bacterial translocation 85. Mice exposed for 30 days to MPs exhibited decreased colonic mucin 2 production and IL-1β along with increased IL-8 and IL-10 levels. These results were accompanied by decreased Firmicutes and increased Bacteroides number as well as enhanced microbiota amino acids metabolism 86. NPs exhibited higher effects compared to MPs in inducing gut macrophage activation in mice, triggering IL-1 dependent inflammation and affecting the gut-brain axis, resulting in microglial activation and T Helper 17 differentiation thus correlating with cognitive and short-term memory impairment 87. Overall, several studies *in vivo* models indicated the ability of MPs and NPs to alter digestive function, with greater effects on the gut microbial composition. The alterations of the microbiota have been correlated to both intestinal and extraintestinal dysfunctions, including hematopoiesis and neural function, possibly indicating MNPs-associated microbiota effects as main players in mediating MNPs effects. Overall, MNPs can cause damage to the digestive system by activating NF-kB and TLR molecular pathways, inducing inflammation, oxidative stress and compromising the intestinal barrier by reducing tight junction proteins, promoting dysbiosis and chronic diseases. Nonetheless, studies are required to confirm and unravel the effects of MNPs on humans.

## Discussion

To date, plastic represents a fundamental part of human life, whose social importance strongly contrasts with its enormous negative environmental and health impact. Increasing evidence is assessing the redox imbalance and toxic effects related to plastics overproduction and over disposal, particularly associated to the continuous release and accumulation on MNPs. Clinical results from Marfella *et al.* are the first clear evidence on the effects of MNPs in human health, possibly setting the basis for the assessment of MNPs exposure as an independent cardiovascular risk factor 56. Nonetheless, evidences, *in vitro* and *in vivo*, are suggesting that the consequences of MNPs in human health could be even more vast, ranging from chronic nervous diseases to gastrointestinal function alterations, as well as fertility and fetus development impairment 88. In recent years, increasing studies are focusing on the accumulation of MNPs other districts also evaluating their effects *in vitro* and *in vivo*. For instance, MPs have been identified via autoptic studies in human lungs, even though their effects in humans have not been assessed 89. In mice models, MPs inhalation has been associated to nasal and lung microbial dysbiosis and experiments in normal human lung epithelial BEAS-2B cells evidenced that MPs can cause cytotoxic and inflammatory effects correlated to reactive oxygen species production and decreased α1-antitrypsin levels, increasing the risk for chronic obstructive pulmonary disease 90,91. MPs can induce senescence of human lung derived A549 and BEAS-2B epithelial cells increasing ROS levels, as well as inducing pro-inflammatory IL-6 and IL-8 cytokines production 91,92,93. In A549 cells, MPs determine the activation of the apoptotic mechanism via Bax, caspase 3, 8 and 9 and cytochrome C upregulation, along with cell cycle S phase arrest 93. Yang *et al.* indicated that MPs can cause redox imbalance and increase levels of MMP9 and surfactant protein A expression in BEAS-2b and human pulmonary alveolar epithelial cells HPAEpiC cells 94. In addition, polystyrene MPs were reported to negatively interact with porcine lung surfactant structure altering its phase behavior, surface tension, and membrane structure, as well as increasing ROS production in lung fluids 95. Irregular polyvinyl chloride NPs *in vitro* treatment of primary human monocytes and monocyte-derived dendritic cells induced high pro-inflammatory cytokines release 96. In human peripheral blood mononuclear cells MPs exposure was able to increase IL-6 and TNF-α production 97. Also, MPs are able to reduce S100A8 expression, thus inducing spleen damage and immune suppression 98. Additionally, studies reported MNPs toxic effects in renal proximal tubular epithelial HK-2. Specifically, MNPs exposure causes oxidative stress, induce apoptosis upregulating Bad and reducing Bcl-2 levels, endoplasmic reticulum stress, autophagy, activation of MAPK and mammalian target of rapamycin (mTOR) signaling and increased extracellular vesicles release 99,100. Moreover, in renal HEK293 cells NPs treatment increased ROS, MDA and LDH levels, while decreasing ATP production and upregulating inflammatory cytokines TNF and IL-6, along with caspase 3 and 9 gene expression 101. Comprehensive, MNPs can cause damage in various systems by triggering common mechanisms such as inflammation, oxidative stress and disruption of cellular barriers. They activate immune responses and impair organs homeostasis, leading to chronic inflammation, cellular dysfunction and microbiome disturbance, contributing to long-term health effects.

Even though different techniques have been developed to detect MNPs in various human tissues, standardized protocols for quantifying MPs in biological samples are still lacking 102,103. There is a substantial gap in epidemiological studies concerning the health effects of MNPs on humans, with most existing research relying on animal studies or *in vitro* experiments. Comprehensive research is crucial to fully understand the health effects and exposure routes of MNPs in humans 103,104. Current methodologies for sampling, isolating, detecting, quantifying, and characterizing MNPs are inadequate, suffering from poor standardization and quality control. Despite this, evidence suggests that MPs are prevalent in food, drinking water, and air. Due to methodological shortcomings and focus on larger particles, existing evaluations probably underestimate human exposure and frequently ignore smaller particles 105,106. Indeed, an important question to be reported is the hypothetic different impact of MPs and NPs in human subjects. Human exposure to NPs is still considered a “scientific challenge”, due to the lack of adequate methods and materials available, as well as a standardization across the analytical procedures utilized 107. Nonetheless, several studies evidenced the peculiar and higher harm of NPs in other biological systems. NPs can penetrate biological barriers more easily due to their small size, thus overpassing cell membranes and the blood-brain barrier 108,109. NPs have a higher surface area to volume ratio, which increases their chemical reactivity and potential for generating ROS, leading to oxidative stress and cellular damage 110. In addition, they can interact with cellular components, leading to genotoxicity and disruption of cellular processes 111. Comparative studies suggest that NPs might pose a greater risk due to their ability to cause systemic effects, including impacting the gut-brain axis and inducing inflammation 87,112. Hopefully, the development of advanced methods to identify and characterize NPs and their effects in human subjects will be soon provided. In the next years, new studies will be able to unravel the uncertainties related to human-plastic relationship, hopefully laying the foundations for a more conscious administration of plastics.

In this scenario, many efforts are focusing on finding adequate means to manage plastics in the best and toxic-less way. In a recent work, three ways to counteract “plastics pandemic” have been investigated, with their potentialities and limitations. Particularly, mechanical recycling, enzymes-based recycling and the production of bioplastics 113. Recycling represents a fundamental part of a circular economy in which plastics would be environmentally affordable 113. Mechanical recycling would be useful to reduce plastics overproduction, still this process could also lead to a down cycling, rendering plastics unrecyclable 113. The development of enzyme-based recycling processes surely presents high potentialities, but is graved by high developmental costs and requires more energy and gases compared to mechanical recycling, along with a high selectivity the requires specific studies and production processes 113,114. As for bioplastics, many studies are focusing on the production of various bio-based types of polymers, such as cellulose or lignin reinforced plastics as well as bacterially biosynthesized bioplastics 115. Bioplastics have been defined as biodegradable materials, able to degrade within months in the environment without producing toxic residues, with the bigger categories represented by polyhydroxyalkanoates and polylactic acid 114.

In the same context, it is acquiring increasing appeal the evaluation of methods to efficiently biodegrade and dispose of plastics already accumulated in human's body, possibly mitigating and dissecting MNPs toxic effects. Studies have demonstrated the potential of the microbiota to degrade MNPs. Specifically, microbes can colonize and introduce hydrophilic functional groups on MNPs surface, thus increasing their susceptibility to degradation via oxidoreductase and hydrolase enzymes. Finally, the products of these reactions can be used as carbon sources by microbes and degraded in oxidative metabolites 116. Interestingly, the administration of probiotics has been shown to improve gut microbiota alterations and intestinal leakage induced by MNPs exposure, along with reduction of inflammatory biomarkers 117. In addition, the intake of probiotics could eliminate common plastic toxic ingredients, including bisphenol A 118. The results of Marfella *et al.* prompted the necessity to define new strategies not only in the prevention but also in the treatment of MNPs toxic effects, due to their ability to increase cardiovascular mortality rates. Hopefully, new methods to reduce the toxic biological effects of accumulated MNPs will be soon provided.

Overall, the further development of these strategies as well as the definition of new ones, along with a more conscious management of plastics production is surely the bases to put an end to plastics pollution crisis and protect environmental and human health.

## Figures and Tables

**Figure 4:**
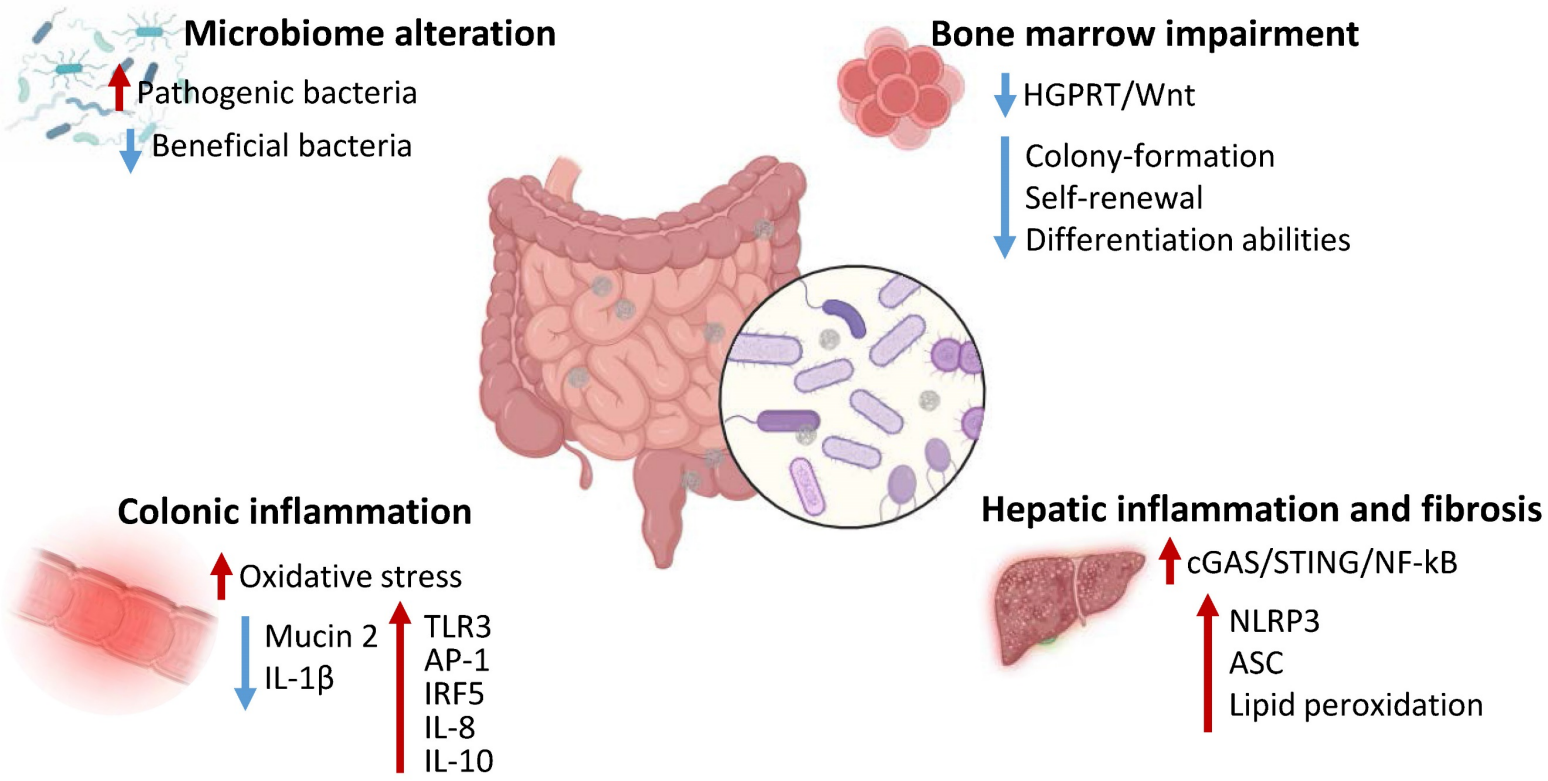
Effects of MNPs on the digestive system and gut microbiota. MNPs determine alterations of gut microbiota, increasing pathogenic bacteria and decreasing beneficial bacterial content, impair bone marrow function inhibiting HGPRT/Wnt pathway and decreasing hematopoietic stem cells function, induce colonic inflammation increasing oxidative stress and inflammatory proteins expression and reducing mucin 2 and IL-1β levels, trigger hepatic inflammation and fibrosis via activation of cGAS/STING/NF-kB pathway and upregulating NLRP3, ASC and lipid peroxidation. HGPRT - hypoxanthine-guanine phosphoribosyltransferase; IL - Interleukin; TLR - Toll-like receptor; AP-1 - activator protein; IRF - interferon regulatory factor; cGAS - cyclic GMP-AMP synthase; STING - Stimulator of interferon genes; NF - nuclear factor; NLRP - NOD-like receptor protein 3; ASC - apoptosis associated speck-like protein.

**Table 1:** Effects of micro- and nanoplastics on human cells and tissues of respiratory, endocrine and immune systems.

System	Sample/Model	Potential MNPs Effects	Reference
Respiratory System	Human lung epithelial cells BEAS-2B	Decrease α1-antitrypsin levels	90,91,93
		Induce IL-6 and IL-8 expression	
	Human alveolar type II epithelial A549 and BEAS-2B	Induce senescence	92,93
		Increase ROS generation and pro-inflammatory state	
	A549	Induce apoptosis increasing BAX, caspase-3, caspase-8, caspase-9 and cytochrome c	93
		Induce cell cycle S phase arrest	
	BEAS-2B and human pulmonary alveolar epithelial cells HPAEpiC	Decrease cell viability and induce redox imbalance	94
		Increase levels of MMP9 and surfactant protein A	
Immunitary System	Human monocytes	Pro-inflammatory cytokines release	96
	Hematopoietic stem cells	Decrease survival and proliferation	81
	Human Peripheral blood mononuclear cell PBMCs	Induce IL-6 and TNF-α production	97
Renal system	Human kidney HK-2 cells	Increase ROS levels	99
		Increase Bad and decrease Bcl-2 protein levels	
		Increase ER stress-and autophagy	
		Increase MAPK and AKT/mTOR signaling pathways	
	HK-2 cells	Increase extracellular vesicles	100
	Embryonic kidney cells HEK 293	Decrease cell viability	101
		Increase oxidative stress	
		Decrease ATP production	
		Increase TNF*,* IL-6, caspase 3 and caspase 9 gene expression	

## Abbreviations

MPs: Microplastics
NPs: Nanoplastics
MNPs: Micro- and nanoplastics
ROS: Reactive Oxygen Species
ATSDR: Agency for Toxic Substances and Disease Registry
PERK: protein kinase R-like endoplasmic reticulum kinase
NF-κB: nuclear factor-κB
TNF-α: tumor necrosis factor-α
IL: interleukin
TLR: toll-like receptor
iNOS: inducible nitric oxide synthase
ZO-1: zonula occludens 1
MBP: myelin basic protein
MOG: myelin oligodendrocyte glycoprotein
ATP: adenosine triphosphate
BMI: Body Mass Index
IUGR: Intrauterine Growth Restriction
HUVECs: human umbilical vein endothelial cells
SOD: superoxide dismutase
MDA: malondialdehyde
JNK: c-Jun NH2-terminal kinase
MAPK: p38 mitogen-activated protein kinase
LDH: lactate dehydrogenase
SDH: succinate dehydrogenase
LPS: lipopolysaccharide
StAR: steroidogenic acute regulatory
MMPs: matrix metalloproteinases
NLRP3: NOD-like receptor protein 3
NOX: NADPH Oxidase
CD: cluster of differentiation
MYH7B: myosin, heavy chain 7B
ANP: atrial natriuretic peptide
BNP: brain natriuretic peptide
COL1A1: Collagen, type I, alpha 1
m6A: N6-methyladenosine
GPx: glutathione peroxidase
mtDNA: mitochondrial DNA
cGAS: cyclic GMP-AMP synthase
STING: stimulator of interferon genes
eNOS: endothelial nitric oxide synthase
SIRT: sirtuin
VEGF: Vascular Endothelial Growth Factor
JAK1: janus kinase 1
STAT: Signal transducer and activator of transcription
TF: tissue factor
AP: activator protein
IRF: interferon regulatory factor
HGPRT: hypoxanthine-guanine phosphoribosyltransferase
ASC: apoptosis associated speck-like protein
Akt: protein kinase B
mTOR: mammalian target of rapamycin
